# Synergistic effect of combined transcranial direct current stimulation/constraint-induced movement therapy in children and young adults with hemiparesis: study protocol

**DOI:** 10.1186/s12887-015-0498-1

**Published:** 2015-11-12

**Authors:** Bernadette Gillick, Jeremiah Menk, Bryon Mueller, Gregg Meekins, Linda E. Krach, Timothy Feyma, Kyle Rudser

**Affiliations:** University of Minnesota, 420 Delaware Street SE, MMC 388, Minneapolis, MN 55455 USA; Biostatistical Design and Analysis Center, University of Minnesota, Minneapolis, MN 55455 USA; Department of Psychiatry, University of Minnesota, Minneapolis, MN 55455 USA; Department of Neurology, University of Minnesota, Minneapolis, MN USA; Courage Kenny Rehabilitation Institute, part of Allina Health, 800 East 28th Street, Minneapolis, MN 55407 USA; Department of Neurology, Gillette Children’s Specialty Healthcare, 200 University Ave E, Saint Paul, MN 55101 USA; Division of Biostatistics, University of Minnesota, Minneapolis, MN USA

**Keywords:** Constraint-induced movement therapy, Non-invasive brain stimulation, Electrical stimulation, Hemiparesis, Pediatrics, Hand function

## Abstract

**Background:**

Perinatal stroke occurs in more than 1 in 2,500 live births and resultant congenital hemiparesis necessitates investigation into interventions which may improve long-term function and decreased burden of care beyond current therapies (http://www.cdc.gov/ncbddd/cp/data.html). Constraint-Induced Movement Therapy (CIMT) is recognized as an effective hemiparesis rehabilitation intervention . Transcranial direct current stimulation as an adjunct treatment to CIMT may potentiate neuroplastic responses and improve motor function. The methodology of a clinical trial in children designed as a placebo-controlled, serial –session, non-invasive brain stimulation trial incorporating CIMT is described here. The primary hypotheses are 1) that no serious adverse events will occur in children receiving non-invasive brain stimulation and 2) that children in the stimulation intervention group will show significant improvements in hand motor function compared to children in the placebo stimulation control group.

**Methods/design:**

A randomized, controlled, double-blinded clinical trial. Twenty children and/or young adults (ages 8–21) with congenital hemiparesis, will be enrolled. The intervention group will receive ten 2-hour sessions of transcranial direct current stimulation combined with constraint-induced movement therapy and the control group will receive sham stimulation with CIMT. The primary outcome measure is safety assessment of transcranial direct current stimulation by physician evaluation, vital sign monitoring and symptom reports. Additionally, hand function will be evaluated using the Assisting Hand Assessment, grip strength and assessment of goals using the Canadian Occupational Performance Measure. Neuroimaging will confirm diagnoses, corticospinal tract integrity and cortical activation. Motor cortical excitability will also be examined using transcranial magnetic stimulation techniques.

**Discussion:**

Combining non-invasive brain stimulation and CIMT interventions has the potential to improve motor function in children with congenital hemiparesis beyond each intervention independently. Such a combined intervention has the potential to benefit an individual throughout their lifetime.

**Trial registration:**

Clinicaltrials.gov, NCT02250092Registered 18 September 2014

## Background

 Based on data and statistics reported by the Center for Disease Control, " Population-based studies from around the world report prevalence estimates of CP ranging from 1.5 to more than 4 per 1,000 live births or children of a defined age range. " Additionally, the lifetime costs of care for an individial diagnosed with cerebral palsy is over 1 million dollars. (http://www.cdc.gov/ncbddd/cp/data.html) [1]. Specific to children, hemiparesis or paralysis on one side of the body, is most commonly caused by an ischemic stroke or vascular disorder, and is often associated with CP [1].

### Significance of diagnosis

Many children with hemiparesis receive rehabilitation, but our current interventions have a limited impact on restoring function. The extended cost and utilization of lengthy formal therapies such as bracing, casting, pharmacologic interventions and surgery can be painful, energy consuming and resource depleting. Treatments such as constraint-induced movement therapy (CIMT) have shown significant improvements in motor function, yet the optimal electrical current dosing in this population has yet to be established [2] Impacting the recovery of a child during critical periods of development with achievement of motor milestones progressing into adulthood is imperative to positively influence an individual who faces the challenges of living with CP. Corticospinal system (CS) development continues postnatally over the first few years of life, and congenital impact or damage to the system before, during or one year after birth can cause detriment to function throughout the individual lifetime [2, 3] Although initially this CS system develops bilaterally in typical development, for those with a perinatal stroke, the integrity of the ipsilateral projections is compromised and control of the limbs occurs from the contralateral hemisphere. This loss is driven by *activity-dependent competition,* between the two hemispheres [4]. As an individual moves and explores the environment through bimanual and unimanual activity, the crossed corticospinal tract integrity gains strength. Typical interhemispheric inhibition is progressively established with potent interaction between the two hemispheric motor cortices and accompanying corticospinal activation and distinct unimanual function.

### Critical barriers to progress in pediatric hemiparesis

If a child incurs a congenital unilateral infarct, the ipsilesional hemisphere may lose the developing crossed-corticospinal tract integrity, while control of bilateral movement may be dominated by the contralesional hemisphere. This adaptation, however, can have a negative impact on the quality and timing of hand function [5, 6]. Surviving neurons in the area of the stroke undergo an imposed dormancy, influenced by the GABA-induced inhibitory effects of the non-lesioned hemisphere; an exaggerated interhemispheric inhibition. No longer does the competition between the two sides exist equally, and an imbalance of use and integrity of CS system occurs. Additionally, in children with congenital hemiparesis who incurred a stroke at or around the time of birth, “developmental disuse” may ensue, wherein the child utilizes the unaffected hand primarily as the preferred hand, with the affected hand utilized in more of an assisting capacity [7, 8]. Without intervention compelling the affected limb to engage in activity, the disability can become continuously more profound and future recovery less possible.

### Promising potential

Encouragingly, the nervous system of a child is plastic [9]."The ability to reestablish a balance between the two hemispheres can therefore be exploited by the following therapeutic interventions of 1) voluntary activation of the involved hemiparetic limbs, 2) electrophysiologic decrease in the exaggerated inhibitory activity of the nonlesioned hemisphere upon the lesioned hemisphere, or 3) direct electrophysiologic activation of the lesioned hemisphere [3] Such potential has been seen, using combined interventions of CIMT and non-invasive brain stimulation (NIBS) and as demonstrated through cortical excitability, imaging and motor mapping studies [10]. This work shows promise for outcomes in the pediatric population with the positive potential to influence function throughout the lifespan. Considerations of safety, cost and efficacy are paramount, with further translational consideration of clinical applicability.

### CIMT

CIMT involves constraining the less-affected or non-paretic upper extremity while activating the clinically more-affected or paretic upper extremity through intensive, structured manual therapy [11]. Day camp models, wherein subjects engage in continuous training while wearing a constraint, have been published previously [12, 13] Such a design allows scheduling of therapy content based on themes and activities within a group setting. Subjects perform activities under the guidance of a therapist or trained/supervised interventionist, with incorporation of established goal setting and attainment.

### NIBS- transcranial direct current stimulation

Transcranial direct current stimulation (tDCS) is currently being investigated as a neuromodulation intervention [14] tDCS has shown beneficial motor behavioral effects in adults [15] and is more cost effective and more portable than another form of NIBS using electromagnetic current called repetitive Transcranial Magnetic Stimulation(rTMS). The mechanism of tDCS involves changing the spontaneous neuronal firing rate and therefore the resting membrane threshold, influencing polarization. Dependent upon the electrode montage and dosing parameters, tDCS involves down-regulating or inhibiting the excitability of the motor cortex in the contralesional hemisphere in an effort to upregulate or disinhibit the ipsilesional hemisphere. Applying *cathodal* tDCS to the contralesional hemisphere has been shown to provide this inhibitory component in adults with stroke, while *anodal* stimulation is excitatory, leading to improved motor function. Importantly, children with diagnoses such as Rasmussen’s Encephalitis, and Schizophrenia have tolerated the use of tDCS well with few minor adverse events such as the sensation of tingling and no reports of pain [16, 17] tDCS has recently been investigated in children with CP and more specifically in our lab in children with unilateral spastic CP with only minor adverse events found [18, 19]. No serious adverse events, such as seizure, have occurred with its use in child or adult populations [14, 20]. Adverse effects of tDCS have been minimized by maintaining current intensity under 2.0 mA and current duration under 20 min. A number of studies have shown that the weak electrical currents applied across the scalp in tDCS are not sufficient to cause tissue damage in the cortex [ 21-23].

### Modes of brain stimulation

As a point of clarity, tDCS should be distinguished from other forms of brain stimulation. For example, traditional electroconvulsive therapy (ECT) induces convulsive activity by delivery of large electrical currents for the treatment of medically refractory depression. In contrast, tDCS influences cortical neurons through the process of neuromodulation, not by the induction of action potentials. Thus, tDCS does not involve the major risks associated with ECT, affecting memory and consciousness. As compared to other non-invasive brain stimulation techniques such as rTMS which carries a low risk of seizure, there have been no reported cases of tDCS inducing seizures either in healthy pediatric or adult human subjects or in child and adult subjects with various neurologic diagnoses[20, 22, 24].

### Change in practice

The significance of this proposed study is that combined CIMT and tDCS could translate research into unprecedented clinical gains in motoric function for individuals with pediatric hemiparesis as 1) the brain during developmental years has a high capacity for plasticity, possibly higher than in adults, 2) neuroplastic change can occur in the injured brain, 3) we are applying a novel neuromodulatory intervention specifically targeted for brain reorganization, and 4) we are combining this intervention with a current clinical behavioral approach (CIMT) that has been shown to influence neuronal excitability and make meaningful clinically important differences.. This could potentially lead to a transformation of rehabilitation practice for this condition Furthermore, even if only a small percentage of the lifetime costs associated with rehabilitating children with hemiparesis were reduced, the aggregate savings would be significant.

Our protocol proposes to non-invasively apply an electrophysiologic intervention with the clinically mature therapy of CIMT. Changes in functional brain connectivity after CIMT have been found in adults and children[10, 25, 26]. However, little research has been performed using non-invasive brain stimulation in pediatric rehabilitation and even less with combined interventions. This project combines a unique form of noninvasive brain stimulation (tDCS) with a behavioral treatment (CIMT) to promote a combined intervention that could achieve higher recovery in pediatric hemiparesis than current treatment alone provides. Both modalities impact excitability of the brain, yet this synergistic approach is novel in children with hemiparesis. The protocol includes measurement of functional and morphologic changes in the brain with neuroimaging and TMS testing to show “proof of principle” data, enriching our understanding of connectivity and responders to intervention. The expected outcomes are no serious adverse effects, improved hand function, and improved functional connectivity of the affected hemisphere.

## Methods/design

### Ethical considerations

This study has been approved by the University of Minnesota and Gillette Children’s Specialty Healthcare Institutional Review Boards. Approval by the University of Minnesota Clinical and Translational Science Institute (UMN CTSI) and its Scientific Review Committee, as well as Center for Magnetic Resonance Research (CMRR), was also obtained. All caregivers and children will be given oral and written information about the study before a request for formal informed consent/assent is signed.

### Objectives

The primary objective of the proposed project is to determine the feasibility of the study design as well as to assess the synergistic effect of combined tDCS/CIMT on safely improving hand motor function in children and young adults between the ages of 8 and 21 with hemiparesis.

### Hypotheses

1. Children with hemiparesis randomized to intervention (tDCS/CIMT) or control (sham tDCS/CIMT) will not display any seizure activity or other serious adverse effect.
2. The intervention group will show greater improvement in paretic hand function (force production, speed, quality of movement) than will controls.
3. The intervention group will show greater improvements in self-reported levels of participation and satisfaction with rehabilitation goals as evidenced by the COPM.

The secondary objective is to examine the influence of combined tDCS/CIMT on brain excitability and reorganization.

### Hypotheses

1. Children in the tDCS/CIMT group will show significantly greater excitability in the ipsilesional hemisphere than will controls.
2. Changes in brain excitability and reorganization will correlate with positive changes in motor function.

### Study design

**Fig. 1:**
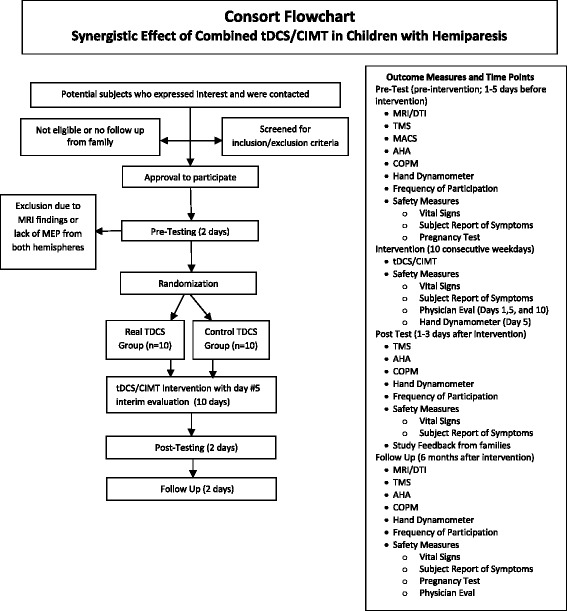
Flowchart of tDCS/CIMT study adapted from the Consolidated Standards of Reporting Trials (CONSORT). AHA: Assisting Hand Assessment, CIMT: Constraint-Induced Movement Therapy, COPM: Canadian Occupational Performance Measure, DTI: Diffusion Tensor Imaging, MRI: Magnetic Resonance Imaging, rs-fMRI: resting-state functional Magnetic Resonance Imaging, tDCS: transcranial Direct Current Stimulation, TMS: Transcranial Magnetic Stimulation

The study will use a randomized, sham-controlled, pretest-posttest-follow up design involving two groups of 10 children with hemiparesis and unilateral infarct or periventricular leukomalacia as confirmed by MRI (total sample size = 20). (Fig. 1). This protocol is registered on clinicaltrials.gov (# NCT02250092)

### Recruitment

We will use our established database of over 200 families to begin recruitment of children with hemiparesis, plus talks at local pediatric facilities, mailings, posting on related websites and newsletters. Incorporation of CIMT provides a motor training component to the study, and families are more willing to participate with the promise of receiving what would be an otherwise expensive intervention now covered by the study.

### Participants- inclusion and exclusion criteria

Children and young adults, ages 8–21, with hemiparesis due to perinatal stroke or periventricular leukomalacia will be recruited for the study based on the following eligibility criteria:

### Inclusion criteria

Subjects will be eligible to participate in the study if the following conditions exist:

1. Hemispheric Stroke or Periventricular Leukomalacia confirmed by most recent MRI or CT radiologic report with resultant congenital hemiparesis
2. ≥ 10 degrees of active motion at the metacarpophalangeal joint
3. No evidence of seizure activity within the last 2 years
4. Presence of a motor evoked potential from at least the contralesional hemisphere if not both hemispheres
5. Ages 8–21 years
6. If ages 8–17, able to give informed assent along with the informed consent of the legal guardian
7. Children who have had surgeries, which may influence motor function e.g.- tendon transfer, will be included, yet surgical history will be documented

### Exclusion criteria

Subjects will be excluded from participation in the study if any of the following conditions exist:

1. Metabolic Disorders
2. Neoplasm
3. Epilepsy
4. Disorders of Cellular Migration and Proliferation
5. Acquired Traumatic Brain Injury
6. Pregnancy
7. Indwelling metal or incompatible medical devices
8. Evidence of skin disease or skin abnormalities
9. Botulinum toxin or Phenol block within the last six-months

### Prior and concomitant therapy

Behavioral therapies including occupational, physical or speech therapies will be allowed both during both the intervention and follow-up period. These therapies will be documented and reported.

### Exit criteria

Subjects will exit the study if any of the following conditions exist:

1. Subject voluntarily withdraws from the study.
2. Subject acquires any of the listed exclusion criteria.
3. Subject completes the protocol.
4. Subject’s well-being, in the opinion of the Investigator, would be compromised by study continuation.
5. Subject experiences a serious adverse event or seizure.
6. Medical monitor and/or IRB recommendation

### Randomization

Each child will be randomized to either the real (intervention) or sham (control) tDCS arm of the study. The randomization will be done by means of sealed envelopes constructed by the study biostatistician using a random number generator. The study coordinator will assign envelopes, in numerical order, to the subjects upon their randomization.

### Blinding

The investigator who performs the testing, the CIMT interventionists and the physician who does the evaluations will be blinded to the treatment arm as will the child/caregiver/family. The study biostatistician, study coordinator and principal investigator (PI) will be unblinded. The following procedure will be employed:

1. The study biostatistician will provide sealed envelopes to the study coordinator.
2. The study coordinator will share the group assignment with the principal investigator in a secure private room, in the absence of the physician, research tester, subject legal guardian and subject. The Medical Monitor to the study will have access to the group assignment.
3. Blinding is applied through designated settings on the tDCS machine of sham or real tDCS. The unblinded investigator who administers the tDCS (PI: BTG) will, during the intervention, switch the setting on the tDCS device to the designated placebo setting, hidden from view of the subject. For the first 30-seconds of either setting, a gradual “ramp-up” sensation of the stimulation occurs. This is built into the machine for both settings. However, for the sham stimulation, the sensation then abates, and not until the end of the 20-minute session does the subject receiving the 30-seconds of “ramp-down” sham once again experience the sensation. The sensation then occurs and “ramps-down” the amperage until the machine turns off.
4. Information on subject group assignment will be logged and stored in a designated locked cabinet at the study coordinators office.

Outcome Assessments. We incorporated the body function/structure and activity/participation domains of the International Classification of Functioning, Disability and Health [2727].

### Primary outcome measure

The Assisting Hand Assessment (AHA) scaled score will be used as the primary outcome measure as a test of unilateral limb dysfunction; it is based on a child’s usual performance in relevant activities. This test uses a standardized video-recorded play session. Activity is assessed on 22 items using a 4-point rating scale. The range of raw scores is 22–88 points, with higher scores indicating better ability. Excellent interrater (0.97) and intrarater (0.99) reliability has been found using this tool and it has been found to have high validity in use with children[28-30]

### Secondary outcome measures

The Canadian Occupational Performance Measure (COPM) is an individualized outcome measure used to detect changes in the self-perception of the client’s performance and satisfaction over time by identifying difficulty in performance of specific activities [31, 32]. The tool is a self-reported ordinal scale score which encompasses domains in impairments, body structure, activity, activity limitations, participation restrictions and environmental factors which an individual experiences. Test-retest reliability has been found to be strong in the domains of performance (r = 0.89) and satisfaction (0.88). Grip strength will also be measured using hand dynamometry[33].

#### Sample size/power

Power calculations for the primary outcome measure AHA scaled score difference between pre- and post-test measurements used the sample size formula for normally distributed statistics with a type I error level of α (2-sided test) and power of (1-β):

$$ n=\frac{V{\left({Z}_{\left(1-\propto /2\right)}+{Z}_{\left(1-\beta \right)}\right)}^2}{\varDelta^2} $$

where Δ denotes the minimal clinically important difference (MCID) to detect, n denotes the sample size per group, and V denotes the variance of the test statistic. For ANCOVA analyses, V = 2(σ^2)(1-ρ^2) where σ^2 is the (average) variability and ρ is the (average) correlation between pre- and post-treatment measurements [34, 35]. Power (1-β) was computed for 10 patients per treatment group across a range of possible values for the correlation with alpha equal to 0.05. These are computed for an MCID of 2.4, and for the rTMS/CIMT study treatment effect of 5.4, which is considered a reasonable estimate for this tDCS exploratory study [36]. The standard deviation from the rTMS/CIMT study was used as an estimate for the power calculations and was equal to 4.7. In addition, 1.5 times the standard deviation of the rTMS/CIMT study was also used in the power calculations to provide power estimates within a conservative standard deviation. The correlation between pre- and post-treatment AHA scores in the rTMS study was 0.97. Based on a correlation of 0.8 and using the conservative standard deviation of 7.1, we will have 80 % power to detect the difference of 5.4 between treatment groups.

#### Study protocol

**Fig. 2:**
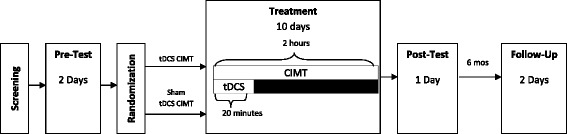
Study design. CIMT, Constraint-Induced Movement Therapy; mos, months; tDCS, Transcranial Direct Current Stimulation

Children will be randomized into two groups: real tDCS/CIMT (intervention) or sham tDCS/CIMT (control). The children will receive 10 continuous weekday sessions of tDCS and CIMT. (Fig. 2) To clarify, we will include only children in whom we can elicit a motor evoked potential (MEP) using TMS applied to the ipsilesional hemisphere. Although by doing so we limit greatly the number of children whom we can include, such conservatism is necessary to proceed in a safe and informed manner with sufficient awareness of the functional topography of each subject’s brain.

##### Rationale for use of tDCS

Our previous research incorporated rTMS, which although promising as an intervention, has limitations especially with children [36, 37]. Our recent results demonstrate not only safety and but also reveal significant improvements in hand function with rTMS combined with constraint-induced movement therapy, yet we foresee the possible greater benefits from using tDCS over this type of non-invasive brain stimulation in pediatric hemiparesis. (REF) First, both of the interventions, tDCS and CIMT, applications of tDCS/CIMT can occur concurrently whereas it is difficult to incorporate the use of rTMS *at the same time* as performing behavioral therapy with the affected upper extremity. Applying tDCS/CIMT simultaneously could optimize neuroplastic principles of concurrent firing of neurons and strengthening of neuronal networks [38, 39]. Second, tDCS may reduce costs. As rTMS can be ten times the current cost of tDCS, cost may become prohibitive when considering rTMS. Additionally, if the application of tDCS reduces the need for additional therapies, a further costs savings could be realized. Third, use of rTMS has resulted in reports, albeit rare, of adverse events such as seizures and syncopal episodes, in both adults and children [40, 41] .

In addition to its economy and portability, tDCS has shown improvements in motor function in adults with and without the concurrent use of CIMT [42, 43]. The rationale for the investigation of tDCS in the pediatric population is that the use of non-invasive brain stimulation could translate more efficiently into clinical applications thus improving the quality of life for children with hemiparesis. And tDCS safety, when using standard guidelines, is supported in the literature as to having common side effects limited to mild and reversible skin irritation. Specific to the tDCS model we are using (Soterix LTE 1×1 tDCS, NY, NY), the design reduces skin irritation by conditioning the skin prior to stimulation and allowing the device operator to incorporate subject feedback and adjust the controls without stopping. This model was specifically chosen for our project because of its adaptability to resistance changes between the surface electrodes and the subject. The model uses two 9 V batteries to administer stimulation. As a safety feature, the unit will automatically reduce the applied voltage so as to maintain a low voltage level. The built-in ramp-up and ramp-down features allow for gradual administration of the current. Another component of this device specifically designed for children is the adjustable strap that allows for optimal contact of the surface electrodes with the skin for reduced irritation.

Safety. Safety testing includes a physician screening with a modified pediatric stroke outcome measure at pretest, interim day 5, posttest and follow-up sessions. Additionally, vital signs are assessed before and after each tDCS/CIMT session and subjects and caregivers complete a subject report of symptoms and a tolerance survey.

Corticospinal tract integrity using MRI Sequences. Resting state fMRI (rs-fMRI) and Diffusion Tensor Imaging (DTI) techniques are becoming alternatives to more traditional task fMRI and structural MRI scans to demonstrate functional and structural connectivity in the brain, particularly in pediatric patients who may be challenged by participating in task based fMRI studies. Our previous work revealed cortical volume differences in children with hemiparesis; further investigation using connectivity metrics may provide a better understanding of the integrity of the corticospinal tract in congenital hemiparesis. Subjects will be scanned at UMN Center for Magnetic Resonance Research on a Siemens 3 T system to obtain a) T1-weighted structural MPRAGE with a 1 mm isotropic spatial resolution; b) High angular resolution multi-band diffusion imaging (HARDI) with whole-brain coverage, 90 slices with 1.5 mm isotropic spatial resolution, b-values of,1000 and 2000 s/mm^2^ with 64 gradient directions per b value, and 16 additional non-diffusion weighted images and c) Resting-state multi-band based functional MRI will be obtained with whole brain coverage, 72 slices with 2 mm isotropic slices, TR = 730 ms, and 500 time points in 6 min. The complete dataset will be obtained in less than 45 min, taking into account time required for setup and comfortable installation of the child in the scanner. Resting-state fMRI data will be processed using independent component analysis (ICA) to identify the sensory-motor area. Activation areas on the baseline scans will be used to refine the site of tDCS and identify seed areas for tractography.

Excitability Measurements using TMS*.* TMS (Magstim, Dyfed, UK) will be used to measure corticospinal excitability in the ipsilesional and contralesional hemispheres. TMS will be delivered with a 70-mm figure-of-eight coil, tangential to head with handle aligned 45 degrees lateral. The assessment will incorporate single-pulse motor threshold, 1 mV and cortical silent period testing. TMS measurements of cortical excitability guided by stereotactic neuronavigation (Brainsight, Rogue Research, Montreal, QC, Canada), will be employed to create a map of the motor cortex from anatomical MRI images obtained at the CMRR. The co-registration of the TMS coil position on the head with the MRI obtained image of the brain anatomy will allow precision in children who have neurologic lesions. All TMS testing will occur in the UMN CTSI by the PI. For this, the child will be seated in a child-sized reclining chair. Surface electromyographic (EMG) electrodes will be attached over the first dorsal interosseous muscles bilaterally, which will record the motor evoked potentials (MEP) resulting from the magnetic stimulation to the M1 region of each hemisphere. Next, the resting motor threshold (RMT) for TMS activation of the target muscle will be determined, and the location of the hotspot defined. If a threshold cannot be elicited within at least the contralesional hemisphere (i.e., no hotspot), the subject will be excluded from the study as position of the tDCS electrodes will be influenced. The RMT will be defined as the lowest amount of stimulation inducing an MEP of at least 50uV on at least 3 of 5 stimulations Cortical excitability will also be tested by administration of 10 TMS pulses, at the minimum stimulus intensity to produce MEPs of 1 mV [44].

CSP will be determined by the subject exerting finger extensor contraction at approximately 30 % of maximum, as guided by a force tracing on a computer display, while a single TMS pulse at an intensity of 150 % of RMT is delivered to the ipsilesional M1 cortex [45, 46]. The resultant CSP will be measured from the onset of the first peak of the MEP to resumption of surface EMG activity with a threshold value of 50 % mean prestimulus amplitude [47].

#### Intervention

##### tDCS/CIMT intervention group

Children randomly assigned to this group will receive 10 sessions of both tDCS and CIMT on concurrent days over a span of two weeks. As supported by results from our previous computerized modeling pilot, the subject will receive tDCS at the motor hotspot at 0.7 mA current intensity for 20 min [19]. In a primary motor cortex (M1)/supraorbital (SO) montage, the anode will be positioned over the ipsilesional supraorbital region and the cathode positioned over contralesional motor hotspot to induce contralesional cortical inhibition. During this 20 min session, the subject will be involved in CIMT for the paretic hand and arm using a sling. The sling will be applied for a 2-hour period, which incorporates the 20 min tDCS session. The CIMT will be given to the subject on an individual basis for two hours each CIMT treatment day by a CIMT-trained therapist/interventionist. The CIMT treatments will be standardized, consisting of shaping activities for function, range of motion and strengthening of the paretic upper extremity. In addition, children will continue to use their paretic limb during functional activities at home and during a caregiver-supervised home program. This combined treatment of tDCS and CIMT will continue until 10 weekday sessions are completed over the two-week period.

##### tDCS/CIMT control group

Children in this group will receive the same procedures and home program as the intervention tDCS/CIMT group but, the tDCS device will be set to a specific placebo setting which extinguishes the current after a 30 s to 1 min ramp-up phase and gradually reintroduces the ramp-down at the end of the 20 min session. This feature allows for placebo control and blinding.

Statistical Analysis. There are 3 analysis populations planned. Intent-to-treat (ITT) will include any subject randomized according to their treatment assignment. Per-protocol (PP) will include randomized subjects without major protocol violations and who were compliant (at least 80 % of planned sessions with their treatment assignment. A detailed list of the major protocol violations warranting exclusion from the PP analysis will be determined prior to trial commencement. The Safety population will include all subjects who receive treatment, according to treatment received. We do not anticipate these groups to differ. The safety profile will use the safety population and be primarily descriptive in nature reporting the number and percentage of adverse events for the two treatment groups and include an aggregate breakdown by severity, seriousness, and frequency (within a patient). Within-group analysis of continuous outcomes comparing posttest to pretest will be done with paired t-tests. Between-group analysis of continuous outcomes comparing the mean change from pretest to posttest between treatment groups will be adjusted for baseline values in the fashion of ANCOVA for added precision. The primary analysis will be based on the ITT population with complementary analyses using the PP population. The association between changes in brain excitability/reorganization and motor function will also be evaluated and based on linear regression. P-values less than 0.05 will be considered statistically significant.

## Discussion

We outline the background and design of a trial with two intervention groups comparing the effects of tDCS on children and young adults with hemiparesis receiving CIMT. The design of this study is predicated upon positive outcomes previously established with CIMT. Additionally, we incorporate consideration of trials we have completed with neuromodulation interventions of repetitive transcranial magnetic stimulation and a tDCS pilot with this population [19, 36, 48]. The proposed study design pursues investigation of a synergistic effect achieved by combining rehabilitation (CIMT) and neuromodulatory (tDCS) interventions. The study hypotheses reflect the importance of safety, feasibility and efficacy surrounding this dual intervention. As non-invasive brain stimulation in pediatric hemiparesis is in a nascent investigational phase, understanding the potential value of such interventions is paramount.

## Abbreviations

AHA: Assisting-Hand Assessment
CP: Cerebral Palsy
CIMT: Constraint-Induced Movement Therapy
COPM: Canadian Occupational Performance Measure
MRI: Magnetic Resonance Imaging
NIBS: Non-Invasive Brain Stimulation
rTMS: Repetitive Transcranial Magnetic Stimulation
tDCS: Transcranial Direct Current Stimulation
TMS: Transcranial Magnetic Stimulation
